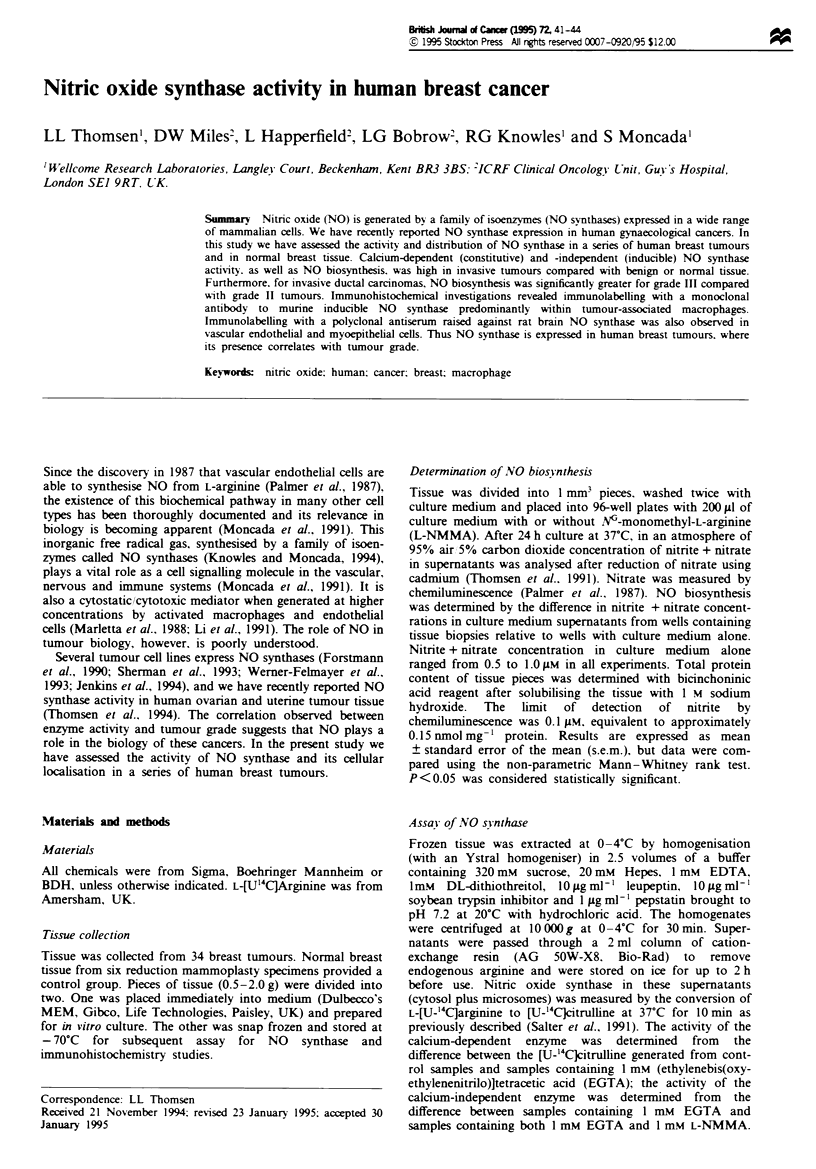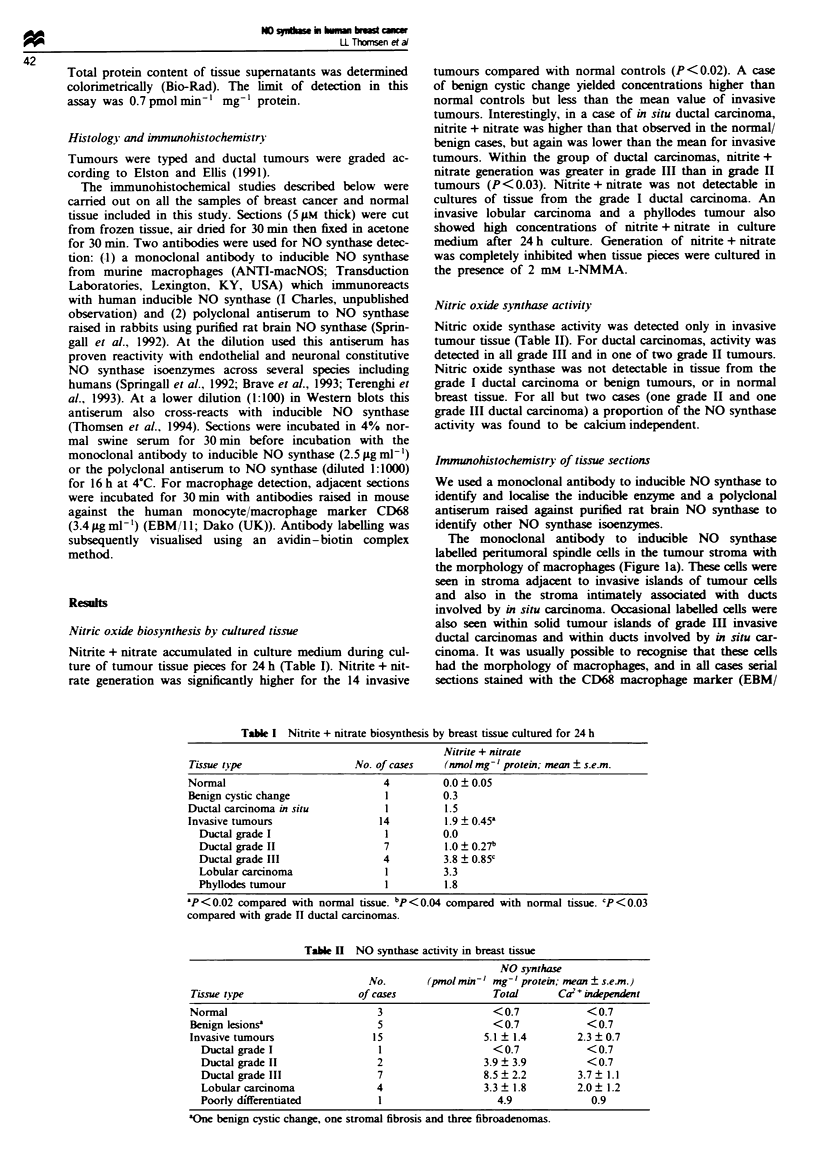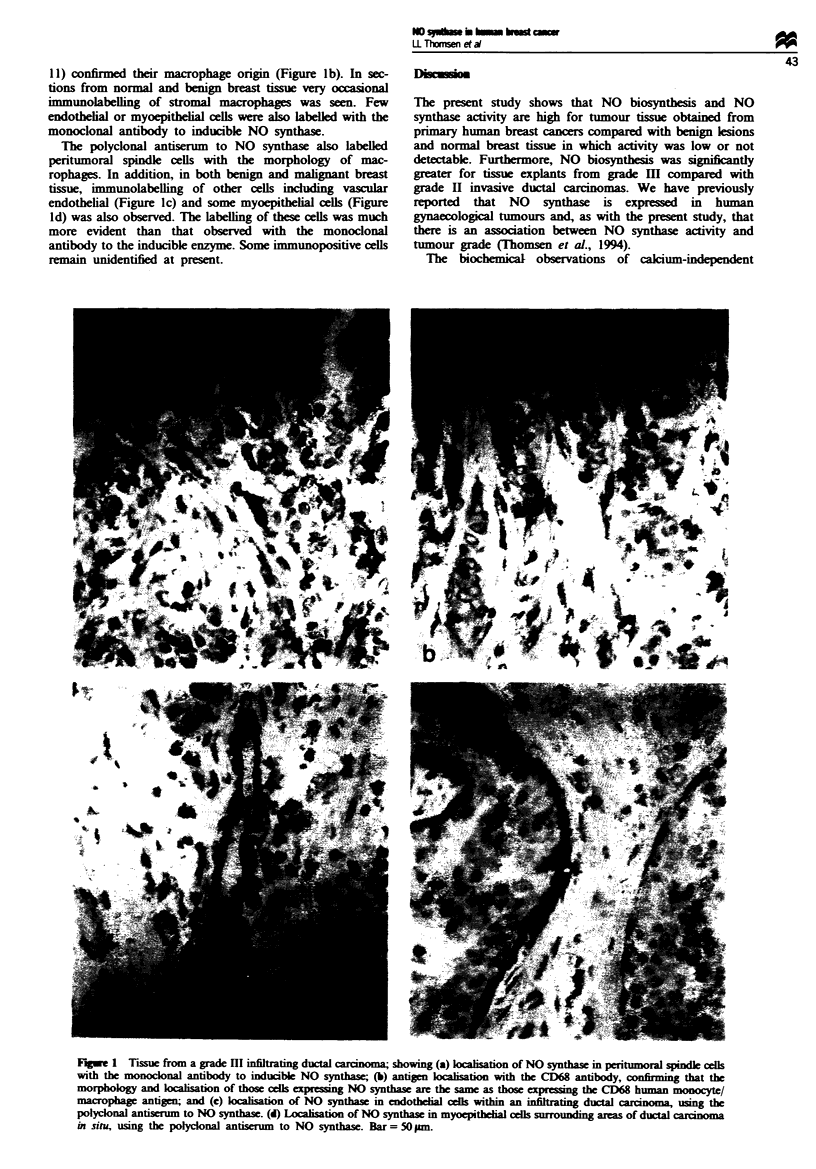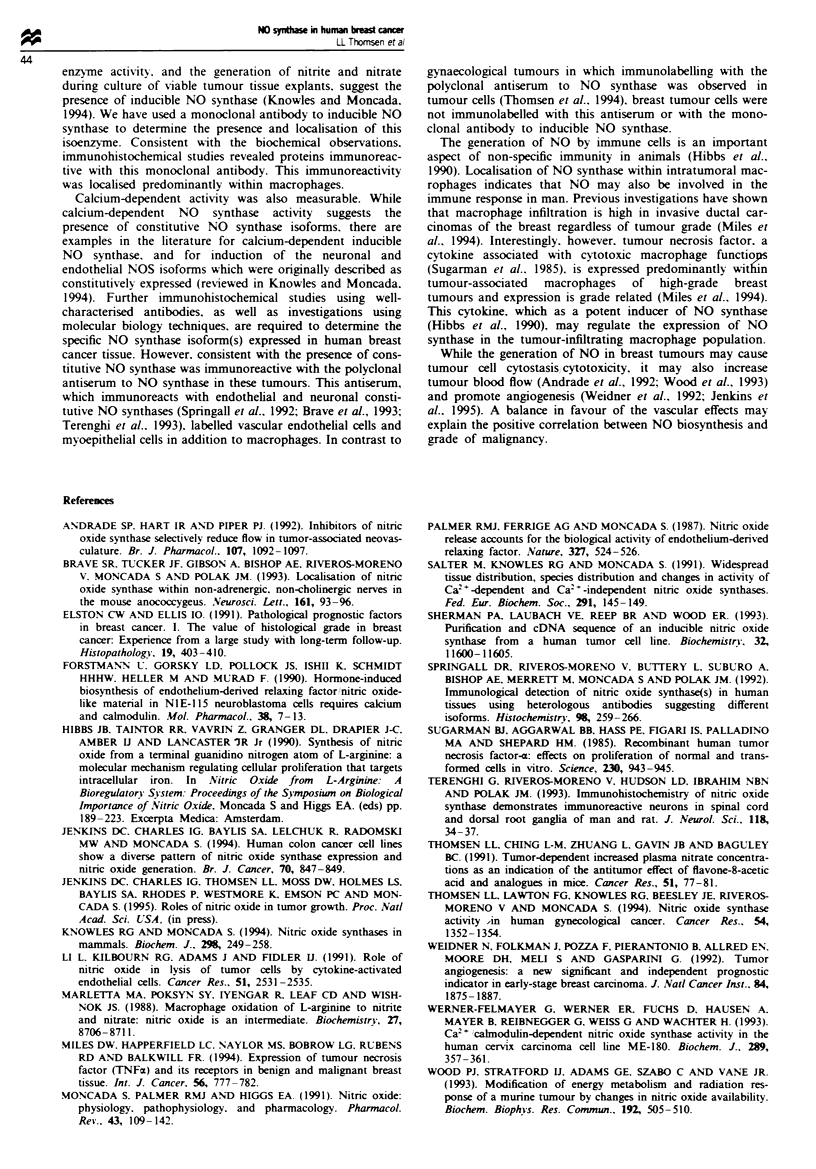# Nitric oxide synthase activity in human breast cancer.

**DOI:** 10.1038/bjc.1995.274

**Published:** 1995-07

**Authors:** L. L. Thomsen, D. W. Miles, L. Happerfield, L. G. Bobrow, R. G. Knowles, S. Moncada

**Affiliations:** Wellcome Research Laboratories, Beckenham, Kent, UK.

## Abstract

**Images:**


© 1995 Stockton Press All rights reserved 0007-0920/95 $12.00

### Nitric oxide synthase activity in human breast cancer

LL Thomsen[1], DW Miles[2], L Happerfield[2], LG Bobrow[2], RG Knowles[1] and S Moncada[1]

[1]*Wellcome Research Laboratories, Langley Court, Beckenham, Kent BR3 3BS;* [2]*ICRF Clinical Oncology Unit, Guy's Hospital, London SE1 9RT, UK.*

**Summary** Nitric oxide (NO) is generated by a family of isoenzymes (NO synthases) expressed in a wide range of mammalian cells. We have recently reported NO synthase expression in human gynaecological cancers. In this study we have assessed the activity and distribution of NO synthase in a series of human breast tumours and in normal breast tissue. Calcium-dependent (constitutive) and -independent (inducible) NO synthase activity, as well as NO biosynthesis, was high in invasive tumours compared with benign or normal tissue. Furthermore, for invasive ductal carcinomas, NO biosynthesis was significantly greater for grade III compared with grade II tumours. Immunohistochemical investigations revealed immunolabelling with a monoclonal antibody to murine inducible NO synthase predominantly within tumour-associated macrophages. Immunolabelling with a polyclonal antiserum raised against rat brain NO synthase was also observed in vascular endothelial and myoepithelial cells. Thus NO synthase is expressed in human breast tumours, where its presence correlates with tumour grade.

**Keywords:** nitric oxide; human; cancer; breast; macrophage

Since the discovery in 1987 that vascular endothelial cells are able to synthesise NO from L-arginine (Palmer *et al.*, 1987), the existence of this biochemical pathway in many other cell types has been thoroughly documented and its relevance in biology is becoming apparent (Moncada *et al.*, 1991). This inorganic free radical gas, synthesised by a family of isoenzymes called NO synthases (Knowles and Moncada, 1994), plays a vital role as a cell signalling molecule in the vascular, nervous and immune systems (Moncada *et al.*, 1991). It is also a cytostatic/cytotoxic mediator when generated at higher concentrations by activated macrophages and endothelial cells (Marletta *et al.*, 1988; Li *et al.*, 1991). The role of NO in tumour biology, however, is poorly understood.

Several tumour cell lines express NO synthases (Forstmann *et al.*, 1990; Sherman *et al.*, 1993; Werner-Felmayer *et al.*, 1993; Jenkins *et al.*, 1994), and we have recently reported NO synthase activity in human ovarian and uterine tumour tissue (Thomsen *et al.*, 1994). The correlation observed between enzyme activity and tumour grade suggests that NO plays a role in the biology of these cancers. In the present study we have assessed the activity of NO synthase and its cellular localisation in a series of human breast tumours.

### Materials and methods

### Materials

All chemicals were from Sigma, Boehringer Mannheim or BDH, unless otherwise indicated. L-[U$^{14}$C]Arginine was from Amersham, UK.

### Tissue collection

Tissue was collected from 34 breast tumours. Normal breast tissue from six reduction mammoplasty specimens provided a control group. Pieces of tissue (0.5-2.0 g) were divided into two. One was placed immediately into medium (Dulbecco's MEM, Gibco, Life Technologies, Paisley, UK) and prepared for *in vitro* culture. The other was snap frozen and stored at $-70$°C for subsequent assay for NO synthase and immunohistochemistry studies.

Correspondence: LL Thomsen
Received 21 November 1994; revised 23 January 1995; accepted 30 January 1995

### Determination of NO biosynthesis

Tissue was divided into 1 mm$^3$ pieces, washed twice with culture medium and placed into 96-well plates with 200 µl of culture medium with or without $N^G$-monomethyl-L-arginine (L-NMMA). After 24 h culture at 37°C, in an atmosphere of 95% air/5% carbon dioxide concentration of nitrite + nitrate in supernatants was analysed after reduction of nitrate using cadmium (Thomsen *et al.*, 1991). Nitrate was measured by chemiluminescence (Palmer *et al.*, 1987). NO biosynthesis was determined by the difference in nitrite + nitrate concentrations in culture medium supernatants from wells containing tissue biopsies relative to wells with culture medium alone. Nitrite + nitrate concentration in culture medium alone ranged from 0.5 to 1.0 µM in all experiments. Total protein content of tissue pieces was determined with bicinchoninic acid reagent after solubilising the tissue with 1 M sodium hydroxide. The limit of detection of nitrite by chemiluminescence was 0.1 µM, equivalent to approximately 0.15 nmol mg$^{-1}$ protein. Results are expressed as mean ± standard error of the mean (s.e.m.), but data were compared using the non-parametric Mann-Whitney rank test. $P<0.05$ was considered statistically significant.

### Assay of NO synthase

Frozen tissue was extracted at 0-4°C by homogenisation (with an Ystral homogeniser) in 2.5 volumes of a buffer containing 320 mM sucrose, 20 mM Hepes, 1 mM EDTA, 1mM DL-dithiothreitol, 10 µg ml$^{-1}$ leupeptin, 10 µg ml$^{-1}$ soybean trypsin inhibitor and 1 µg ml$^{-1}$ pepstatin brought to pH 7.2 at 20°C with hydrochloric acid. The homogenates were centrifuged at 10 000 *g* at 0-4°C for 30 min. Supernatants were passed through a 2 ml column of cation-exchange resin (AG 50W-X8, Bio-Rad) to remove endogenous arginine and were stored on ice for up to 2 h before use. Nitric oxide synthase in these supernatants (cytosol plus microsomes) was measured by the conversion of L-[U-$^{14}$C]arginine to [U-$^{14}$C]citrulline at 37°C for 10 min as previously described (Salter *et al.*, 1991). The activity of the calcium-dependent enzyme was determined from the difference between the [U-$^{14}$C]citrulline generated from control samples and samples containing 1 mM (ethylenebis(oxyethylenenitrilo)]tetracetic acid (EGTA); the activity of the calcium-independent enzyme was determined from the difference between samples containing 1 mM EGTA and samples containing both 1 mM EGTA and 1 mM L-NMMA.

Total protein content of tissue supernatants was determined colorimetrically (Bio-Rad). The limit of detection in this assay was 0.7 pmol $min^{-1}$ $mg^{-1}$ protein.

*Histology and immunohistochemistry*

Tumours were typed and ductal tumours were graded according to Elston and Ellis (1991).

The immunohistochemical studies described below were carried out on all the samples of breast cancer and normal tissue included in this study. Sections (5 μM thick) were cut from frozen tissue, air dried for 30 min then fixed in acetone for 30 min. Two antibodies were used for NO synthase detection: (1) a monoclonal antibody to inducible NO synthase from murine macrophages (ANTI-macNOS; Transduction Laboratories, Lexington, KY, USA) which immunoreacts with human inducible NO synthase (I Charles, unpublished observation) and (2) polyclonal antiserum to NO synthase raised in rabbits using purified rat brain NO synthase (Springall *et al.*, 1992). At the dilution used this antiserum has proven reactivity with endothelial and neuronal constitutive NO synthase isoenzymes across several species including humans (Springall *et al.*, 1992; Brave *et al.*, 1993; Terenghi *et al.*, 1993). At a lower dilution (1:100) in Western blots this antiserum also cross-reacts with inducible NO synthase (Thomsen *et al.*, 1994). Sections were incubated in 4% normal swine serum for 30 min before incubation with the monoclonal antibody to inducible NO synthase (2.5 μg $ml^{-1}$) or the polyclonal antiserum to NO synthase (diluted 1:1000) for 16 h at 4°C. For macrophage detection, adjacent sections were incubated for 30 min with antibodies raised in mouse against the human monocyte/macrophage marker CD68 (3.4 μg $ml^{-1}$) (EBM/11; Dako (UK)). Antibody labelling was subsequently visualised using an avidin–biotin complex method.

### Results

*Nitric oxide biosynthesis by cultured tissue*

Nitrite + nitrate accumulated in culture medium during culture of tumour tissue pieces for 24 h (Table I). Nitrite + nitrate generation was significantly higher for the 14 invasive tumours compared with normal controls ($P<0.02$). A case of benign cystic change yielded concentrations higher than normal controls but less than the mean value of invasive tumours. Interestingly, in a case of *in situ* ductal carcinoma, nitrite + nitrate was higher than that observed in the normal/benign cases, but again was lower than the mean for invasive tumours. Within the group of ductal carcinomas, nitrite + nitrate generation was greater in grade III than in grade II tumours ($P<0.03$). Nitrite + nitrate was not detectable in cultures of tissue from the grade I ductal carcinoma. An invasive lobular carcinoma and a phyllodes tumour also showed high concentrations of nitrite + nitrate in culture medium after 24 h culture. Generation of nitrite + nitrate was completely inhibited when tissue pieces were cultured in the presence of 2 mM L-NMMA.

*Nitric oxide synthase activity*

Nitric oxide synthase activity was detected only in invasive tumour tissue (Table II). For ductal carcinomas, activity was detected in all grade III and in one of two grade II tumours. Nitric oxide synthase was not detectable in tissue from the grade I ductal carcinoma or benign tumours, or in normal breast tissue. For all but two cases (one grade II and one grade III ductal carcinoma) a proportion of the NO synthase activity was found to be calcium independent.

*Immunohistochemistry of tissue sections*

We used a monoclonal antibody to inducible NO synthase to identify and localise the inducible enzyme and a polyclonal antiserum raised against purified rat brain NO synthase to identify other NO synthase isoenzymes.

The monoclonal antibody to inducible NO synthase labelled peritumoral spindle cells in the tumour stroma with the morphology of macrophages (Figure 1a). These cells were seen in stroma adjacent to invasive islands of tumour cells and also in the stroma intimately associated with ducts involved by *in situ* carcinoma. Occasional labelled cells were also seen within solid tumour islands of grade III invasive ductal carcinomas and within ducts involved by *in situ* carcinoma. It was usually possible to recognise that these cells had the morphology of macrophages, and in all cases serial sections stained with the CD68 macrophage marker (EBM/

**Table I** Nitrite + nitrate biosynthesis by breast tissue cultured for 24 h

| *Tissue type* | *No. of cases* | *Nitrite + nitrate (nmol $mg^{-1}$ protein; mean ± s.e.m.* |
|---|---|---|
| Normal | 4 | 0.0 ± 0.05 |
| Benign cystic change | 1 | 0.3 |
| Ductal carcinoma *in situ* | 1 | 1.5 |
| Invasive tumours | 14 | 1.9 ± 0.45[a] |
| Ductal grade I | 1 | 0.0 |
| Ductal grade II | 7 | 1.0 ± 0.27[b] |
| Ductal grade III | 4 | 3.8 ± 0.85[c] |
| Lobular carcinoma | 1 | 3.3 |
| Phyllodes tumour | 1 | 1.8 |

[a]$P<0.02$ compared with normal tissue. [b]$P<0.04$ compared with normal tissue. [c]$P<0.03$ compared with grade II ductal carcinomas.

**Table II** NO synthase activity in breast tissue

| *Tissue type* | *No. of cases* | *NO synthase (pmol $min^{-1}$ $mg^{-1}$ protein; mean ± s.e.m.)* Total | $Ca^{2+}$ *independent* |
|---|---|---|---|
| Normal | 3 | <0.7 | <0.7 |
| Benign lesions[a] | 5 | <0.7 | <0.7 |
| Invasive tumours | 15 | 5.1 ± 1.4 | 2.3 ± 0.7 |
| Ductal grade I | 1 | <0.7 | <0.7 |
| Ductal grade II | 2 | 3.9 ± 3.9 | <0.7 |
| Ductal grade III | 7 | 8.5 ± 2.2 | 3.7 ± 1.1 |
| Lobular carcinoma | 4 | 3.3 ± 1.8 | 2.0 ± 1.2 |
| Poorly differentiated | 1 | 4.9 | 0.9 |

[a]One benign cystic change, one stromal fibrosis and three fibroadenomas.

11) confirmed their macrophage origin (Figure 1b). In sections from normal and benign breast tissue very occasional immunolabelling of stromal macrophages was seen. Few endothelial or myoepithelial cells were also labelled with the monoclonal antibody to inducible NO synthase.

The polyclonal antiserum to NO synthase also labelled peritumoral spindle cells with the morphology of macrophages. In addition, in both benign and malignant breast tissue, immunolabelling of other cells including vascular endothelial (Figure 1c) and some myoepithelial cells (Figure 1d) was also observed. The labelling of these cells was much more evident than that observed with the monoclonal antibody to the inducible enzyme. Some immunopositive cells remain unidentified at present.

### Discussion

The present study shows that NO biosynthesis and NO synthase activity are high for tumour tissue obtained from primary human breast cancers compared with benign lesions and normal breast tissue in which activity was low or not detectable. Furthermore, NO biosynthesis was significantly greater for tissue explants from grade III compared with grade II invasive ductal carcinomas. We have previously reported that NO synthase is expressed in human gynaecological tumours and, as with the present study, that there is an association between NO synthase activity and tumour grade (Thomsen *et al.*, 1994).

The biochemical observations of calcium-independent

**Figure 1** Tissue from a grade III infiltrating ductal carcinoma; showing (**a**) localisation of NO synthase in peritumoral spindle cells with the monoclonal antibody to inducible NO synthase; (**b**) antigen localisation with the CD68 antibody, confirming that the morphology and localisation of those cells expressing NO synthase are the same as those expressing the CD68 human monocyte/macrophage antigen; and (**c**) localisation of NO synthase in endothelial cells within an infiltrating ductal carcinoma, using the polyclonal antiserum to NO synthase. (**d**) Localisation of NO synthase in myoepithelial cells surrounding areas of ductal carcinoma *in situ*, using the polyclonal antiserum to NO synthase. Bar = 50 μm.

enzyme activity, and the generation of nitrite and nitrate during culture of viable tumour tissue explants, suggest the presence of inducible NO synthase (Knowles and Moncada, 1994). We have used a monoclonal antibody to inducible NO synthase to determine the presence and localisation of this isoenzyme. Consistent with the biochemical observations, immunohistochemical studies revealed proteins immunoreactive with this monoclonal antibody. This immunoreactivity was localised predominantly within macrophages.

Calcium-dependent activity was also measurable. While calcium-dependent NO synthase activity suggests the presence of constitutive NO synthase isoforms, there are examples in the literature for calcium-dependent inducible NO synthase, and for induction of the neuronal and endothelial NOS isoforms which were originally described as constitutively expressed (reviewed in Knowles and Moncada, 1994). Further immunohistochemical studies using well-characterised antibodies, as well as investigations using molecular biology techniques, are required to determine the specific NO synthase isoform(s) expressed in human breast cancer tissue. However, consistent with the presence of constitutive NO synthase was immunoreactive with the polyclonal antiserum to NO synthase in these tumours. This antiserum, which immunoreacts with endothelial and neuronal constitutive NO synthases (Springall *et al.*, 1992; Brave *et al.*, 1993; Terenghi *et al.*, 1993), labelled vascular endothelial cells and myoepithelial cells in addition to macrophages. In contrast to gynaecological tumours in which immunolabelling with the polyclonal antiserum to NO synthase was observed in tumour cells (Thomsen *et al.*, 1994), breast tumour cells were not immunolabelled with this antiserum or with the monoclonal antibody to inducible NO synthase.

The generation of NO by immune cells is an important aspect of non-specific immunity in animals (Hibbs *et al.*, 1990). Localisation of NO synthase within intratumoral macrophages indicates that NO may also be involved in the immune response in man. Previous investigations have shown that macrophage infiltration is high in invasive ductal carcinomas of the breast regardless of tumour grade (Miles *et al.*, 1994). Interestingly, however, tumour necrosis factor, a cytokine associated with cytotoxic macrophage functions (Sugarman *et al.*, 1985), is expressed predominantly within tumour-associated macrophages of high-grade breast tumours and expression is grade related (Miles *et al.*, 1994). This cytokine, which as a potent inducer of NO synthase (Hibbs *et al.*, 1990), may regulate the expression of NO synthase in the tumour-infiltrating macrophage population.

While the generation of NO in breast tumours may cause tumour cell cytostasis/cytotoxicity, it may also increase tumour blood flow (Andrade *et al.*, 1992; Wood *et al.*, 1993) and promote angiogenesis (Weidner *et al.*, 1992; Jenkins *et al.*, 1995). A balance in favour of the vascular effects may explain the positive correlation between NO biosynthesis and grade of malignancy.

### References

ANDRADE SP, HART IR AND PIPER PJ. (1992). Inhibitors of nitric oxide synthase selectively reduce flow in tumor-associated neovasculature. *Br. J. Pharmacol.*, **107**, 1092–1097.

BRAVE SR, TUCKER JF, GIBSON A, BISHOP AE, RIVEROS-MORENO V, MONCADA S AND POLAK JM. (1993). Localisation of nitric oxide synthase within non-adrenergic, non-cholinergic nerves in the mouse anococcygeus. *Neurosci. Lett.*, **161**, 93–96.

ELSTON CW AND ELLIS IO. (1991). Pathological prognostic factors in breast cancer. I. The value of histological grade in breast cancer: Experience from a large study with long-term follow-up. *Histopathology*, **19**, 403–410.

FORSTMANN U, GORSKY LD, POLLOCK JS, ISHII K, SCHMIDT HHHW, HELLER M AND MURAD F. (1990). Hormone-induced biosynthesis of endothelium-derived relaxing factor/nitric oxide-like material in N1E-115 neuroblastoma cells requires calcium and calmodulin. *Mol. Pharmacol.*, **38**, 7–13.

HIBBS JB, TAINTOR RR, VAVRIN Z, GRANGER DL, DRAPIER J-C, AMBER IJ AND LANCASTER JR Jr (1990). Synthesis of nitric oxide from a terminal guanidino nitrogen atom of L-arginine: a molecular mechanism regulating cellular proliferation that targets intracellular iron. In *Nitric Oxide from L-Arginine: A Bioregulatory System: Proceedings of the Symposium on Biological Importance of Nitric Oxide*, Moncada S and Higgs EA. (eds) pp. 189–223. Excerpta Medica: Amsterdam.

JENKINS DC, CHARLES IG, BAYLIS SA, LELCHUK R, RADOMSKI MW AND MONCADA S. (1994). Human colon cancer cell lines show a diverse pattern of nitric oxide synthase expression and nitric oxide generation. *Br. J. Cancer*, **70**, 847–849.

JENKINS DC, CHARLES IG, THOMSEN LL, MOSS DW, HOLMES LS, BAYLIS SA, RHODES P, WESTMORE K, EMSON PC AND MONCADA S. (1995). Roles of nitric oxide in tumor growth. *Proc. Natl Acad. Sci. USA*, (in press).

KNOWLES RG AND MONCADA S. (1994). Nitric oxide synthases in mammals. *Biochem. J.*, **298**, 249–258.

LI L, KILBOURN RG, ADAMS J AND FIDLER IJ. (1991). Role of nitric oxide in lysis of tumor cells by cytokine-activated endothelial cells. *Cancer Res.*, **51**, 2531–2535.

MARLETTA MA, POKSYN SY, IYENGAR R, LEAF CD AND WISHNOK JS. (1988). Macrophage oxidation of L-arginine to nitrite and nitrate: nitric oxide is an intermediate. *Biochemistry*, **27**, 8706–8711.

MILES DW, HAPPERFIELD LC, NAYLOR MS, BOBROW LG, RUBENS RD AND BALKWILL FR. (1994). Expression of tumour necrosis factor (TNFα) and its receptors in benign and malignant breast tissue. *Int. J. Cancer*, **56**, 777–782.

MONCADA S, PALMER RMJ AND HIGGS EA. (1991). Nitric oxide: physiology, pathophysiology, and pharmacology. *Pharmacol. Rev.*, **43**, 109–142.

PALMER RMJ, FERRIGE AG AND MONCADA S. (1987). Nitric oxide release accounts for the biological activity of endothelium-derived relaxing factor. *Nature*, **327**, 524–526.

SALTER M, KNOWLES RG AND MONCADA S. (1991). Widespread tissue distribution, species distribution and changes in activity of $Ca^{2+}$-dependent and $Ca^{2+}$-independent nitric oxide synthases. *Fed. Eur. Biochem. Soc.*, **291**, 145–149.

SHERMAN PA, LAUBACH VE, REEP BR AND WOOD ER. (1993). Purification and cDNA sequence of an inducible nitric oxide synthase from a human tumor cell line. *Biochemistry*, **32**, 11600–11605.

SPRINGALL DR, RIVEROS-MORENO V, BUTTERY L, SUBURO A, BISHOP AE, MERRETT M, MONCADA S AND POLAK JM. (1992). Immunological detection of nitric oxide synthase(s) in human tissues using heterologous antibodies suggesting different isoforms. *Histochemistry*, **98**, 259–266.

SUGARMAN BJ, AGGARWAL BB, HASS PE, FIGARI IS, PALLADINO MA AND SHEPARD HM. (1985). Recombinant human tumor necrosis factor-α: effects on proliferation of normal and transformed cells in vitro. *Science*, **230**, 943–945.

TERENGHI G, RIVEROS-MORENO V, HUDSON LD, IBRAHIM NBN AND POLAK JM. (1993). Immunohistochemistry of nitric oxide synthase demonstrates immunoreactive neurons in spinal cord and dorsal root ganglia of man and rat. *J. Neurol. Sci.*, **118**, 34–37.

THOMSEN LL, CHING L-M, ZHUANG L, GAVIN JB AND BAGULEY BC. (1991). Tumor-dependent increased plasma nitrate concentrations as an indication of the antitumor effect of flavone-8-acetic acid and analogues in mice. *Cancer Res.*, **51**, 77–81.

THOMSEN LL, LAWTON FG, KNOWLES RG, BEESLEY JE, RIVEROS-MORENO V AND MONCADA S. (1994). Nitric oxide synthase activity in human gynecological cancer. *Cancer Res.*, **54**, 1352–1354.

WEIDNER N, FOLKMAN J, POZZA F, PIERANTONIO B, ALLRED EN, MOORE DH, MELI S AND GASPARINI G. (1992). Tumor angiogenesis: a new significant and independent prognostic indicator in early-stage breast carcinoma. *J. Natl Cancer Inst.*, **84**, 1875–1887.

WERNER-FELMAYER G, WERNER ER, FUCHS D, HAUSEN A, MAYER B, REIBNEGGER G, WEISS G AND WACHTER H. (1993). $Ca^{2+}$/calmodulin-dependent nitric oxide synthase activity in the human cervix carcinoma cell line ME-180. *Biochem. J.*, **289**, 357–361.

WOOD PJ, STRATFORD IJ, ADAMS GE, SZABO C AND VANE JR. (1993). Modification of energy metabolism and radiation response of a murine tumour by changes in nitric oxide availability. *Biochem. Biophys. Res. Commun.*, **192**, 505–510.